# Baseline glucose-to-lymphocyte ratio predicts nivolumab outcomes in advanced non-small cell lung cancer: a multicenter retrospective study

**DOI:** 10.3389/fimmu.2026.1714533

**Published:** 2026-02-12

**Authors:** Mustafa Ersoy, Umut Kefeli, Devrim Cabuk, Ertugrul Bayram, Duygu Bayir, Oktay Bozkurt, Muslih Ürün, Ramazan Coşar, Teoman Sakalar, Emel Mutlu, Muhammet Cengiz, Elif Sahin, Pervin Sancı, Canan Yıldız, Erdem Kolemen, Ayse Cengiz, Gozde Agdas, Erkam Kocaaslan, Ezgi Turkoglu, Sedat Yıldırım, Berrak Mermeit, Anil Karakayalı, Hayati Arvas, Mehmet Mutlu, Sedat Biter, Havva Yeşil Çinkir, Mehmet H. Yücel

**Affiliations:** 1 Department of Internal Medicine, Division of Medical Oncology, Kutahya Health Sciences University, Kutahya, Türkiye; 2 Kocaeli Universitesi Tip Fakultesi, Izmit, Türkiye; 3 Cukurova Universitesi Tip Fakultesi, Adana, Türkiye; 4 Eskisehir Osmangazi Universitesi Tip Fakultesi, Eskişehir, Türkiye; 5 Erciyes Universitesi Tip Fakultesi, Kayseri, Türkiye; 6 Van Yuzuncu Yil Universitesi, Van, Türkiye; 7 Afyon Kocatepe Universitesi Tip Fakultesi, Afyonkarahisar, Türkiye; 8 Türkiye Cumhuriyeti (TC) Saglik Bakanligi Kahramanmaras Necip Fazil Sehir Hastanesi, Dulkadiroğlu, Türkiye; 9 Türkiye Cumhuriyeti (TC) Saglik Bakanligi Corum Erol Olcok Egitim ve Arastirma Hastanesi, Çorum, Türkiye; 10 Türkiye Cumhuriyeti (TC) Saglik Bakanligi Kocaeli Sehir Hastanesi, Izmit, Türkiye; 11 Van Egitim ve Arastirma Hastanesi, Van, Türkiye; 12 Türkiye Cumhuriyeti (TC) Saglik Bakanligi Pendik Egitim ve Arastirma Hastanesi, Pendik, Türkiye; 13 Kartal Dr Lutfi Kirdar Sehir Hastanesi, Istanbul, Türkiye; 14 Van Yuzuncu Yil Universitesi Tip Fakultesi, Van, Türkiye; 15 Dicle Universitesi Tip Fakultesi, Diyarbakır, Türkiye; 16 Gaziantep Universitesi Tip Fakultesi, Gaziantep, Türkiye; 17 Department of Internal Medicine, Division of Medical Oncology, Istanbul Medipol Universitesi Tip Fakultesi, Beykoz, Türkiye

**Keywords:** biomarkers, glucose-to-lymphocyte ratio, immune checkpoint inhibitors, immunometabolism, nivolumab, non-small cell lung cancer, prognosis, survival

## Abstract

**Background:**

Biomarkers guiding immunotherapy in non-small cell lung cancer (NSCLC) are limited. The glucose-to-lymphocyte ratio (GLR), integrating metabolic and immune status, has shown prognostic value in several cancers but has not been systematically evaluated in patients receiving PD-1 blockade.

**Methods:**

We retrospectively analyzed 837 patients with advanced or metastatic NSCLC treated with nivolumab across 21 oncology centers in Turkey (2015–2025). Baseline GLR was calculated from fasting glucose and absolute lymphocyte counts. Overall survival (OS) and progression-free survival (PFS) were estimated using Kaplan–Meier and compared with log-rank tests. Multivariate Cox regression models identified independent predictors. Receiver operating characteristic (ROC) analysis determined the optimal GLR cut-off for OS, which was subsequently applied to PFS analyses for consistency.

**Results:**

The optimal GLR cut-off for mortality was ≥70.76 (AUC = 0.635, 95% CI: 0.597–0.674; p < 0.001). Patients with GLR <70.76 achieved significantly longer OS (median 24.1 vs 9.6 months; p < 0.001) and PFS (9.7 vs 5.8 months; p < 0.001) compared with those with GLR ≥70.76. In multivariate analysis, high GLR and poor ECOG performance status independently predicted worse OS. Prior thoracic radiotherapy was associated with improved outcomes.

**Conclusion:**

Baseline GLR is a practical, cost-effective biomarker that independently predicts OS in advanced NSCLC patients treated with nivolumab. Elevated GLR likely reflects metabolic dysfunction and impaired immune reserve, both unfavorable for PD-1 blockade efficacy. Prospective studies are warranted to validate GLR and define its role in clinical decision-making.

## Introduction

Lung cancer remains the leading cause of cancer-related mortality worldwide, accounting for nearly one in five cancer deaths (1). Non-small cell lung cancer (NSCLC) constitutes approximately 85% of all lung cancer cases, and the prognosis of advanced disease remains poor despite advances in systemic therapies (2). In recent years, immune checkpoint inhibitors (ICIs), particularly agents targeting the programmed death-1 (PD-1)/programmed death ligand-1 (PD-L1) pathway, have transformed the therapeutic landscape of NSCLC (3). Nivolumab, a fully human IgG4 anti–PD-1 antibody, has demonstrated durable clinical benefit and is now a standard second-line treatment for patients with advanced NSCLC following platinum-based chemotherapy (4–6).

Despite these advances, only a subset of patients derives long-term benefit from PD-1 blockade, and reliable predictive biomarkers remain limited (7). PD-L1 expression and tumor mutational burden (TMB) are the most widely investigated markers, but both have significant limitations in terms of predictive accuracy, feasibility, and reproducibility (8, 9). Therefore, the identification of simple, cost-effective, and reproducible biomarkers that reflect host immune status and metabolic fitness is a major unmet need in immuno-oncology.

Several inflammation-based indices derived from routine blood tests, such as the neutrophil-to-lymphocyte ratio and platelet-to-lymphocyte ratio, have been explored as prognostic markers in cancer and immunotherapy settings (10). While these indices primarily reflect systemic inflammatory burden, they do not directly capture the metabolic conditions that critically influence immune cell function (11). In contrast, biomarkers that integrate metabolic status with immune competence may provide additional insight into host-related determinants of response to immune checkpoint blockade (12).

Glucose metabolism is closely intertwined with immune function (13). Tumor cells rely on increased glucose uptake and glycolysis (the Warburg effect), which not only supports tumor proliferation but also creates an immunosuppressive microenvironment by depleting nutrients and accumulating lactate (14). Concurrently, lymphocytes play a central role in antitumor immunity and the efficacy of ICIs, with peripheral blood lymphocyte counts serving as surrogates of host immune competence (15). Reduced lymphocyte availability has consistently been linked to poor outcomes across malignancies (16).

The glucose-to-lymphocyte ratio (GLR), derived from routine laboratory tests, integrates systemic metabolic status with immune reserve. Previous studies have reported its prognostic value in gastrointestinal and hepatocellular cancers (17–20). However, its role as a predictive biomarker in NSCLC patients treated with immunotherapy remains largely unexplored.

The present multicenter study aimed to evaluate the prognostic and predictive significance of baseline GLR in a large real-world cohort of advanced NSCLC patients treated with nivolumab. By investigating the association between GLR and survival outcomes, we sought to determine whether this accessible biomarker can provide additional clinical guidance in patient selection and decision-making for PD-1 blockade.

## Methods

### Study design and setting

This was a multicenter, retrospective cohort study conducted across 21 oncology centers in Turkey. Patients with advanced or metastatic non-small cell lung cancer (NSCLC) who received nivolumab between January 2015 and January 2025 were included. The study was performed in accordance with the Declaration of Helsinki and approved by the local ethics committees of all participating centers.

### Patient population

Eligible patients were ≥18 years old with histologically confirmed advanced or metastatic NSCLC who had received at least one cycle of nivolumab following platinum-based chemotherapy. Availability of baseline laboratory data, including fasting serum glucose and absolute lymphocyte count (within 14 days before nivolumab initiation), and adequate follow-up for survival analysis were required. Patients with other active malignancies, missing key data, or who discontinued nivolumab after the first cycle for non-medical reasons, defined as patient refusal, administrative or reimbursement-related issues, logistical barriers, or loss to follow-up, rather than treatment-related toxicity or disease progression, were excluded.

### Data collection

Demographic, clinical, and treatment-related data were extracted from electronic medical records. Variables included age, sex, body mass index (BMI), smoking status, comorbidities (diabetes, hypertension, COPD, heart failure, arrhythmia, cerebrovascular disease), ECOG performance status, histology (adenocarcinoma, squamous, others), PD-L1 expression, and metastatic sites (brain, liver, adrenal). PD-L1 assessment was performed according to institutional practice using different commercially available assays and antibody clones across participating centers, without centralized pathology review. As a result, inter-assay variability and a substantial proportion of missing PD-L1 data were present, reflecting real-world clinical conditions. Details of prior systemic therapies, thoracic radiotherapy, and treatment line of nivolumab were also collected.

### Laboratory and imaging assessment

Baseline laboratory values were obtained from routine blood tests performed within 14 days prior to nivolumab initiation. The glucose–lymphocyte ratio (GLR) was calculated as:

GLR = Fasting serum glucose (mg/dL)/Absolute lymphocyte count (10³/µL).

Imaging assessments, including computed tomography (CT) or positron emission tomography (PET-CT), were performed at baseline and repeated at intervals according to institutional practice. Disease progression was determined from radiology reports; where available, progression was defined according to the immune Response Evaluation Criteria in Solid Tumors (iRECIST) to account for atypical immune-related response patterns. Although imaging schedules varied across centers, follow-up assessments were most commonly performed every 8–12 weeks in accordance with routine nivolumab monitoring practices.

### Clinical outcomes

The primary endpoint was progression-free survival (PFS), defined as the time from nivolumab initiation to the first documented disease progression or death from any cause. Patients without an event were censored at the date of last follow-up.

The secondary endpoint was overall survival (OS), defined as the time from nivolumab initiation to death from any cause. Patients alive at data cut-off were censored at their last available follow-up.

Follow-up time was estimated using the reverse Kaplan–Meier method.

### Statistical analysis

All analyses were conducted using IBM SPSS Statistics for Windows, Version 23.0 (IBM Corp. Armonk, NY, USA). Continuous variables were summarized as mean ± standard deviation (SD) or median (interquartile range, IQR), and categorical variables as frequencies and percentages.

- ROC curve analysis was performed to evaluate the predictive performance of GLR for mortality, and the Youden index was used to determine the optimal cut-off.
- OS and PFS were estimated by Kaplan–Meier, with comparisons between groups made using the log-rank test.
- Variables with p < 0.10 in univariate analysis, along with clinically relevant covariates, were entered into multivariable Cox regression models. Hazard ratios (HRs) and 95% confidence intervals (CIs) were calculated.
- The proportional hazards assumption was tested using Schoenfeld residuals and time-dependent covariates.
- A two-tailed p-value < 0.05 was considered statistically significant.

### Ethical approval

The study protocol was approved by the Non-Interventional Research Ethics Committee of Bezmialem Vakif University (decision number: E-54022451-050.04-206870; meeting date: 30 June 2025; meeting number: 2025/311). Informed consent was waived due to the retrospective design.

## Results

### Patient characteristics

A total of 837 patients with advanced or metastatic NSCLC were included in the study, of whom 807 had complete data for GLR-based analyses (**Table 1**). The median age was 64 years (range, 31–89), and the majority of patients were male (86.6%) with a history of smoking. Adenocarcinoma (44.8%) and squamous cell carcinoma (46.1%) were the predominant histological subtypes. When stratified by GLR, baseline demographic characteristics—including age, sex, body mass index, and smoking status—were well balanced between groups. In contrast, patients with elevated GLR more frequently had diabetes mellitus and poorer ECOG performance status. PD-L1 expression was available for 382 patients, and the comparison of PD-L1 distribution between GLR groups was limited to this subset, with no significant difference observed; more than half of the overall cohort had missing PD-L1 data. Metastatic burden, including brain, liver, and adrenal metastases, as well as prior treatment exposure, were comprehensively detailed and compared between groups. Overall, these data demonstrate that GLR stratification was not driven by gross imbalances in baseline characteristics, supporting the validity of subsequent outcome analyses.

**Table 1:** Baseline sociodemographic, clinical, and tumor characteristics stratified by glucose-to-lymphocyte ratio (GLR).

Variables	GLR < 70.76 (n = 390)	GLR ≥ 70.76 (n = 417)	p-value
Demographics			
Age, years, median (IQR)	64 (58–71)	64 (58–70)	0.763
Sex, male, n (%)	339 (86.9)	362 (86.3)	0.962
BMI, kg/m², median (IQR)	25.3 (22.4–28.4)	24.6 (21.8–27.6)	0.928
Smoking (never / current / former), n (%)			0.461
Never	64 (16.4)	69 (16.5)	
Current	161 (41.2)	157 (37.6)	
Former	158 (40.5)	187 (44.8)	
Comorbidities			
Diabetes mellitus, n (%)	66 (16.9)	115 (27.5)	<0.001
Hypertension, n (%)	137 (35.1)	153 (36.7)	0.644
COPD, n (%)	117 (30.0)	128 (30.7)	0.890
Heart failure, n (%)	21 (5.4)	36 (8.6)	0.07
Arrhythmia, n (%)	26 (6.6)	41 (9.8)	0.09
Cerebrovascular disease, n (%)	6 (1.5)	12 (2.9)	0.19
Disease characteristics			
Histology (Adeno / SCC / Other), n (%)			0.962
Adenocarcinoma	176 (47.8)	181 (42.9)	
Squamous cell carcinoma	178 (43.0)	198 (47.0)	
Other	36 (9.2)	42 (10.1)	
ECOG PS (0 / 1 / ≥2), n (%)			**0.016**
0	85 (27.6)	86 (22.1)	
1	272 (61.4)	265 (60.7)	
≥2	33 (11.0)	64 (17.3)	
PD-L1 status n (%)			0.242
Negative	83 (21.2)	62(14.9)	
1–50%	73 (18.7)	75(18.0)	
>50%	42(10.7)	47(11.3)	
Missing	193(49.4)	233(55.9)	
Metastatic status (de novo), n (%)	269 (68.8)	281 (67.4)	0.67
Brain metastasis, n (%)	79 (20.2)	82 (19.6)	0.833
Liver metastasis, n (%)	62 (15.9)	76 (18.2)	0.448
Adrenal metastasis, n (%)	78 (20.0)	100 (23.9)	0.364
Treatment history			
Prior platinum-based chemotherapy, n (%)	374 (95.7)	398 (95.4)	0.85
Prior thoracic/mediastinal RT, n (%)	143 (36.6)	157 (37.6)	0.773
Line of nivolumab therapy, median (IQR)	2 (2–3)	2 (2–3)	0.884
Baseline laboratory values			
Baseline glucose, mg/dL, median (IQR)	96 (89–104)	123 (112–138)	<0.001
Baseline lymphocyte count, /µL, median (IQR)	1760 (1420–2110)	1080 (820–1360)	<0.001

Values are presented as median (interquartile range, IQR) or number (percentage), as appropriate. P-values for continuous variables were derived using the Mann–Whitney U test due to non-normal distributions; categorical variables were compared using the χ² test or Fisher’s exact test, as appropriate. BMI, body mass index; COPD, chronic obstructive pulmonary disease; ECOG PS, Eastern Cooperative Oncology Group performance status; RT, radiotherapy; PD-L1, programmed death-ligand 1; SCC, squamous cell carcinoma; GLR, glucose-to-lymphocyte ratio.

### Predictive value of GLR

**Figure 1:**
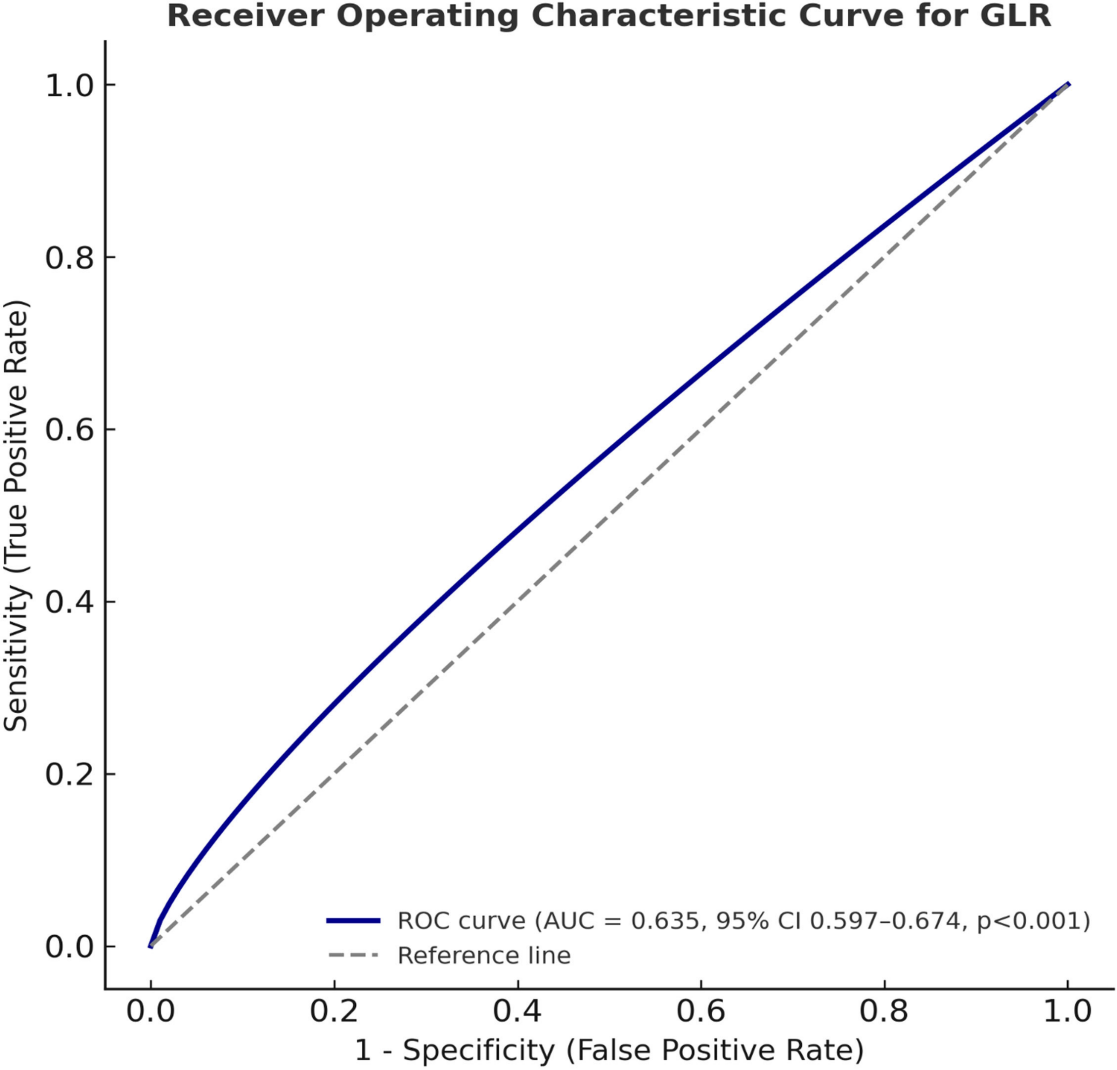
Receiver operating characteristic (ROC) curve evaluating the predictive performance of the glucose-to-lymphocyte ratio (GLR) for overall survival in patients treated with nivolumab. The area under the curve (AUC) was 0.635 (95% CI: 0.597–0.674, p < 0.001). The optimal cut-off identified by the Youden index was GLR ≥70.76, corresponding to a sensitivity of 61.3% and specificity of 61.2%.

ROC analysis identified an optimal GLR cut-off of ≥70.76 for mortality prediction (AUC = 0.635, 95% CI: 0.597–0.674; p < 0.001; **Table 2**, **Figure 1**). This threshold yielded 61.3% sensitivity and 61.2% specificity.

**Table 2:** Predictive value of glucose-to-lymphocyte ratio for mortality by ROC analysis.

Variable	AUC	95% CI	Cut-off	Sensitivity (%)	Specificity (%)	p-value
Glucose-to-Lymphocyte Ratio	0.635	0.597–0.674	≥70.76	61.3	61.2	<0.001

Analysis performed using receiver operating characteristic (ROC) curves. AUC, Area under the curve; CI, Confidence interval.

#### OS

**Figure 2:**
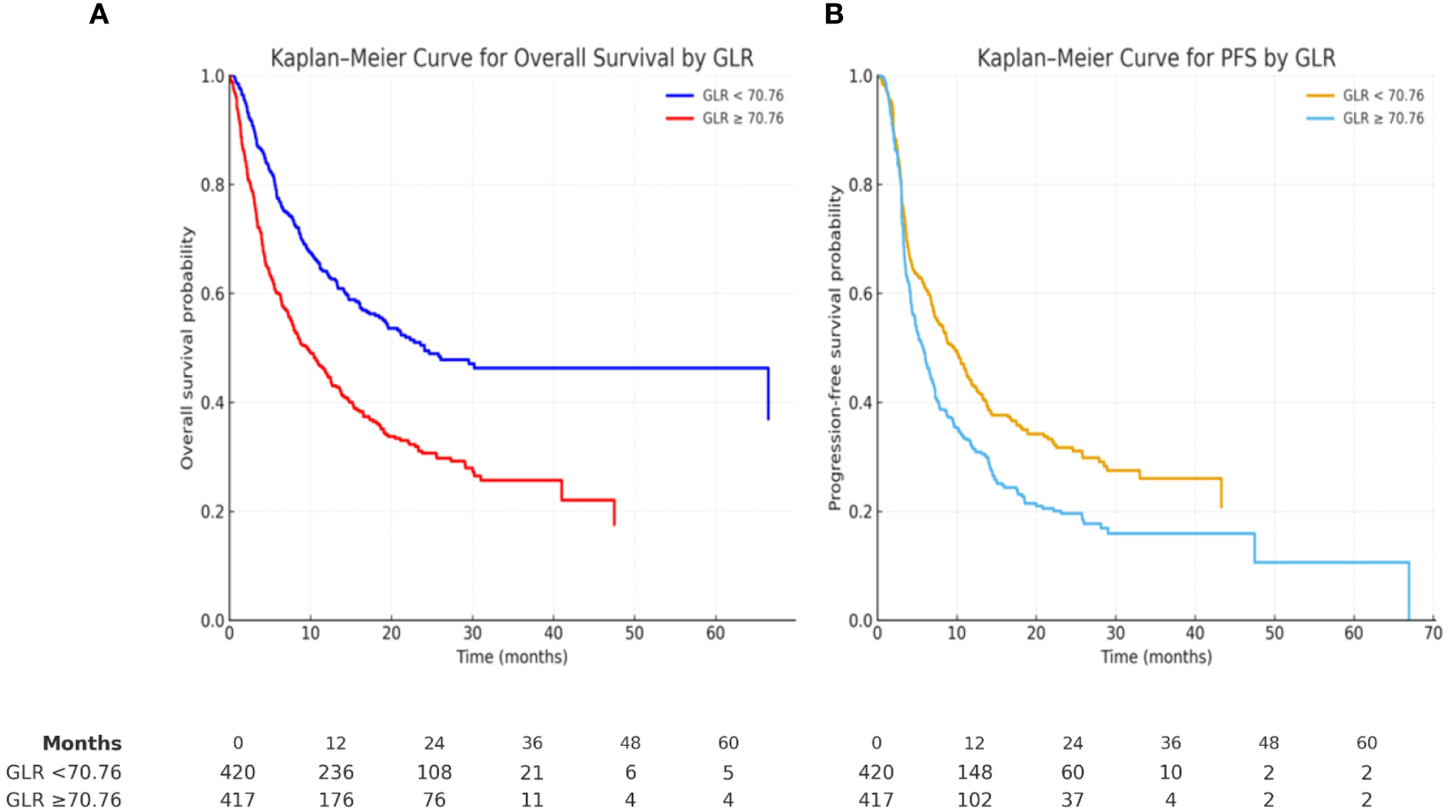
Kaplan–Meier survival curves stratified by glucose-to-lymphocyte ratio (GLR). **(A)** Overall survival (OS). Patients with GLR < 70.76 showed significantly longer survival compared with GLR ≥ 70.76 (log-rank p < 0.001).**(B)** Progression-free survival (PFS). Patients with GLR < 70.76 had significantly longer PFS compared with GLR ≥ 70.76 (log-rank p < 0.001).

The median OS for the cohort was 14.0 months (95% CI: 11.7–16.2). The estimated 2- and 5-year OS rates were 36% and 24%, respectively (**Table 3**). Patients with GLR <70.76 achieved significantly longer OS compared with those with GLR ≥70.76 (median 24.1 vs 9.6 months; log-rank p < 0.001; **Figure 2A**). Subgroup analyses showed that OS differed significantly by ECOG performance status (p < 0.001) and prior thoracic radiotherapy (p = 0.001), but not by age, sex, smoking status, histology, or PD-L1 expression.

**Table 3:** Overall survival comparisons by patient characteristics.

Variable	2-year OS (%)	5-year OS (%)	Median OS, months (95% CI)	p-value
Overall	36	24	14.0 (11.7–16.2)	—
**Age**				0.923
≤65	37	17	14.3(11.3–15.3)	
>65	35	33	13.7(11.1–16.3)	
**Sex**				0.167
Male	35	24	13.3(11.3–15.3)	
Female	40	40	19.3 (12.0–26.6)	
**Histology**				0.091
Adenocarcinoma	41	20	17.2 (12.2–22.3)	
Squamous cell carcinoma (SCC)	30	27	12.0 (9.6–14.4)	
NOS	47	16	20.5 (6.7–34.2)	
AdenoSCC	45	45	16.9 (NR-NR)	
Other	25	25	6.9 (1.7–12.1)	
**Smoking**				0.784
Never	37	19	15.7 (9.1–22.3)	
Current	33	27	13.6 (9.9–17.3)	
Former	37	29	13.8 (11.0–16.6)	
**ECOG**				<0.001
0	48	43	29.5 (NR-NR)	
1	36	21	15.7 (12.9–18.5)	
2	16	16	4.8 (3.2–6.4)	
3	13	13	2.8 (0.5–5.1)	
**Thoracic RT prior/during nivolumab**				0.001
No	32	17	12.0 (9.6–14.4)	
Yes	42	35	18.9 (11.4–26.4)	
**PD-L1 expression**				0.587
Negative	44	15	21.1 (10.7–31.5)	
1–50	40	40	17.2 (9.9–24.6)	
>50	49	39	29.1 (16.3–42.0)	
**Glucose-to-Lymphocyte Ratio**				<0.001
<70.76	47	30	24.1 (9.5–38.6)	
≥70.76	28	18	9.6 (7.4–11.8)	

OS comparisons were performed using Kaplan–Meier survival analysis and the log-rank test. OS, Overall survival; CI, Confidence interval; ECOG, Eastern Cooperative Oncology Group; NOS, Not otherwise specified; RT, Radiotherapy; NR, Not reached. Bold values indicate statistical significance (p < 0.05).

#### PFS

The median PFS was 6.9 months (95% CI: 6.0–7.8). The 2- and 5-year PFS rates were 22% and 9%, respectively (**Table 4**). Patients with GLR <70.76 had significantly longer PFS compared with those with GLR ≥70.76 (median 9.7 vs 5.8 months; log-rank p < 0.001; **Figure 2B**). PFS was also associated with ECOG performance status (p < 0.001) and prior thoracic radiotherapy (p = 0.002). Histology showed a borderline association (p = 0.085).

**Table 4:** Progression-free survival comparisons by patient characteristics.

Variable	2-year PFS (%)	5-year PFS (%)	Median PFS, months (95% CI)	p-value
Overall	22	9	6.9 (6.0–7.8)	—
**Age**				0.265
≤65	20	—	6.6 (5.8–7.5)	
>65	24	14	7.3 (5.7–8.9)	
**Sex**				0.499
Male	23	10	6.8 (5.9–7.6)	
Female	15	—	8.6 (6.6–10.6)	
**Histology**				0.085
Adenocarcinoma	23	—	7.2 (5.8–9.7)	
SCC	20	20	6.8 (5.9–8.5)	
NOS	36	—	4.9 (1.1–10.8)	
AdenoSCC	32	32	12.3 (NE)	
Other	9	—	3.6 (0.8–6.5)	
**Smoking**				0.687
Never	18	9	6.5 (4.1–9.0)	
Current	22	22	7.2 (5.8–8.5)	
Former	23	5	6.7 (5.3–8.1)	
**ECOG**				<0.001
0	33	20	8.4 (3.2–13.6)	
1	21	8	7.50 (6.2–8.8)	
2	11	—	3.3 (2.8–3.8)	
3	—	—	1.9 (0.8–3.1)	
**Thoracic RT prior/during nivolumab**				0.002
No	20	—	5.2 (4.2–6.3)	
Yes	25	17	9.6 (7.9–11.4)	
**PD-L1 expression**				0.101
Negative	23	23	5.2 (3.3–7.1)	
1–50	22	—	7.0 (4.8–9.1)	
>50	29	23	11.1 (3.2–19.2)	
**Glucose-to-Lymphocyte Ratio**				<0.001
<70.76	28	21	9.7 (7.7–11.7)	
≥70.76	17	4	5.8 (4.9–6.8)	

PFS comparisons were performed using Kaplan–Meier survival analysis and the log-rank test. “—” indicates 5-year estimates not calculable due to small number at risk. PFS, Progression-free survival; CI, Confidence interval; ECOG, Eastern Cooperative Oncology Group; NOS, Not otherwise specified; RT, Radiotherapy; SCC, Squamous cell carcinoma; NE, Not estimable. Bold values indicate statistical significance (p < 0.05).

### GLR quartile analysis

In an exploratory quartile-based analysis, overall survival differed significantly across baseline GLR quartiles (log-rank χ² = 42.861, df = 3, *p* < 0.001). Median OS progressively decreased with increasing GLR, from 24.1 months in the lowest quartile to 7.3 months in the highest quartile. Similarly, progression-free survival also differed significantly across GLR quartiles (log-rank χ² = 17.680, df = 3, *p* = 0.001), with median PFS declining from 8.9 months in the lowest quartile to 4.4 months in the highest quartile. These findings demonstrate a consistent inverse association between baseline GLR levels and survival outcomes.

### Multivariate cox regression analyses

**Figure 3:**
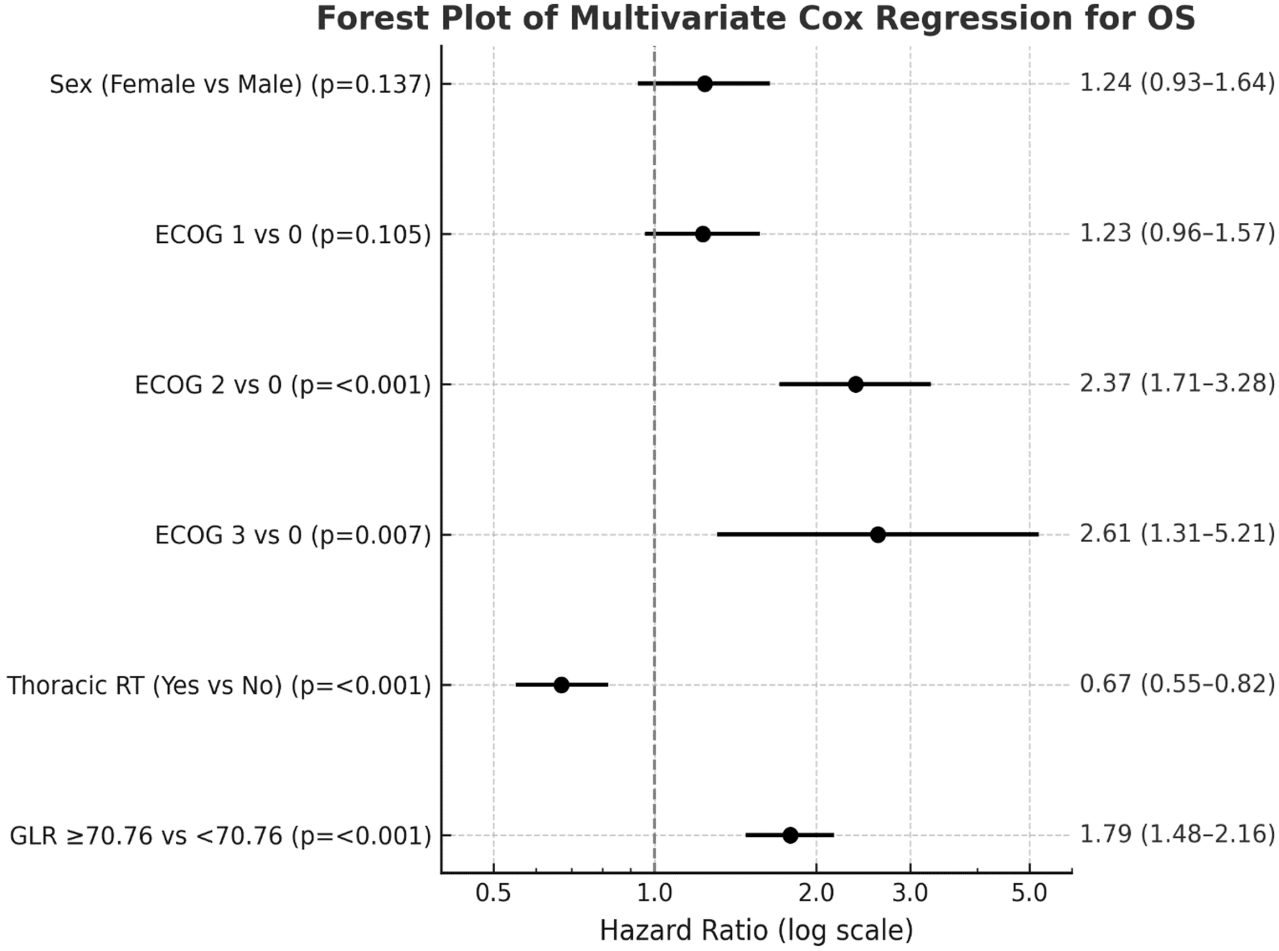
Forest plot from multivariate Cox regression analysis for overall survival (OS). ECOG performance status and GLR ≥70.76 were independent predictors of worse OS, whereas prior thoracic radiotherapy was associated with improved OS. Hazard ratios (HRs) are presented with 95% confidence intervals (CIs).

In multivariate models (**Table 5**, **6**; **Figure 3**), ECOG performance status remained the most consistent independent predictor of both OS and PFS. Compared with ECOG 0, patients with ECOG 2 and 3 had significantly increased risks of death (HR 2.37, 95% CI: 1.71–3.28; p < 0.001 and HR 2.61, 95% CI: 1.31–5.21; p = 0.007, respectively). A baseline GLR ≥70.76 was independently associated with worse OS (HR 1.79, 95% CI: 1.48–2.16; p < 0.001), but did not reach statistical significance for PFS (HR 1.22, 95% CI: 0.94–1.58; p = 0.136). Prior thoracic radiotherapy was independently associated with improved OS (HR 0.67, 95% CI: 0.55–0.82; p < 0.001) and PFS (HR 0.74, 95% CI: 0.56–0.97; p = 0.031). Sex, histology, and PD-L1 expression were not retained as independent predictors.

**Table 5:** Multivariate cox regression analysis of mortality risk.

Variable	HR (95% CI)	p-value
**Sex**		0.137
Male	Reference	
Female	1.24 (0.93–1.64)	
**ECOG performance status**		<0.001
0	Reference	—
1	1.23 (0.96–1.57)	0.105
2	2.37 (1.71–3.28)	<0.001
3	2.61 (1.31–5.21)	0.007
**Thoracic RT prior/during nivolumab**		<0.001
No	Reference	
Yes	0.67 (0.55–0.82)	
**Glucose-to-Lymphocyte Ratio**		<0.001
<70.76	Reference	
≥70.76	1.79 (1.48–2.16)	

Multivariate Cox regression model included variables significant in the univariate analysis. HR, Hazard ratio; CI, Confidence interval; ECOG, Eastern Cooperative Oncology Group; RT, Radiotherapy. Model statistics: 2 Log Likelihood = 5594.96, p < 0.001. Bold values indicate statistical significance (p < 0.05).

**Table 6:** Multivariate cox regression analysis of progression risk.

Variable	HR (95% CI)	p-value
**Histology**		0.537
Adenocarcinoma	Reference	—
SCC	1.07 (0.82–1.41)	0.617
NOS	1.07 (0.54–2.14)	0.842
AdenoSCC	1.05 (0.51–2.19)	0.893
Other	1.71 (0.94–3.10)	0.078
**ECOG performance status**		<0.001
0	Reference	—
1	1.13 (0.81–1.57)	0.487
2	1.65 (1.01–2.70)	0.045
3	12.75 (4.79–33.97)	<0.001
**Thoracic RT prior/during nivolumab**		0.031
No	Reference	
Yes	0.74 (0.56–0.97)	
**PD-L1 expression**		0.234
Negative	Reference	—
1–50	0.95 (0.71–1.26)	0.707
>50	0.75 (0.53–1.05)	0.095
**Glucose-to-Lymphocyte Ratio**		0.136
<70.76	Reference	
≥70.76	1.22 (0.94–1.58)	

Multivariate Cox regression model included variables significant in the univariate analysis. HR, Hazard ratio; CI, Confidence interval; ECOG, Eastern Cooperative Oncology Group; RT, Radiotherapy; SCC, Squamous cell carcinoma; NOS, Not otherwise specified. Model statistics: 2 Log Likelihood = 2602.08, p < 0.001. Bold values indicate statistical significance (p < 0.05).

## Discussion

In this large multicenter cohort of advanced NSCLC patients treated with nivolumab, we demonstrated that the GLR, derived from routine blood tests, is an independent predictor of OS. Patients with elevated GLR experienced significantly shorter OS, whereas the prognostic value of GLR for PFS did not reach statistical significance in multivariate analysis (p = 0.136). This finding mirrors the results of pivotal immunotherapy trials such as CheckMate-017 and CheckMate-057, in which PD-1 blockade significantly improved OS but showed only modest or nonsignificant effects on PFS (5, 6). These data suggest that GLR may capture biological features related to long-term immunological fitness, consistent with the OS-driven efficacy profile of nivolumab.

Several factors may explain why the prognostic value of GLR was stronger for OS than for PFS in our adjusted analyses. PFS is highly dependent on the timing and consistency of radiological assessments, which varied across participating centers in this retrospective cohort and may dilute true biomarker effects. In addition, immune checkpoint inhibitors exhibit unique response kinetics—including delayed tumor regression, immune-related inflammatory flares, and pseudoprogression—that can result in early radiologic progression despite the potential for durable benefit (21). These phenomena disproportionately affect PFS but have less influence on OS, which captures the cumulative long-term survival advantage derived from sustained immune activation (22). Importantly, this pattern mirrors findings from pivotal immunotherapy trials where nivolumab and other PD-1/PD-L1 inhibitors demonstrated significant improvements in OS despite minimal or nonsignificant effects on PFS (5, 6, 23, 24). This broader evidence base reinforces the biological plausibility of GLR being more strongly associated with OS than with PFS.

Mechanistically, the association between elevated GLR and poorer outcomes may reflect a broader disruption of immunometabolic homeostasis within the tumor microenvironment. Cancer cells undergo metabolic reprogramming characterized by high glycolytic activity and excessive glucose consumption, resulting in nutrient competition that restricts substrates essential for effective T-cell function (25). This metabolic imbalance is further compounded by lactate accumulation and microenvironmental acidification, both of which suppress antitumor immune responses (26). Elevated GLR may therefore serve as a systemic surrogate of this disturbed metabolic–immune interface, representing a physiological state in which tumor-driven metabolic demands exceed host immune capacity, thereby limiting the efficacy of PD-1 blockade (27).

From a biological perspective, high GLR reflects the combined impact of hyperglycemia and lymphopenia—two conditions independently associated with impaired antitumor immunity. Hyperglycemia has been shown to accelerate CD8^+^ T-cell exhaustion, increase PD-1 expression, and promote immunosuppressive cellular subsets, while reduced lymphocyte counts limit the pool of effector T cells available for reinvigoration by immune checkpoint inhibition (28–30). In addition, dysregulated glucose metabolism may activate the PI3K/AKT/mTOR signaling axis, impairing memory T-cell differentiation and favoring short-lived effector phenotypes (31, 32). Collectively, these mechanisms provide a biologically plausible explanation for the association between elevated GLR and inferior survival outcomes in patients receiving nivolumab.

Our results are consistent with previous studies reporting the prognostic significance of GLR in gastrointestinal and hepatocellular cancers (19, 20). By extending these findings to lung cancer in the context of immunotherapy, we highlight the broader relevance of this biomarker. Importantly, GLR differs from other systemic indices such as the neutrophil-to-lymphocyte ratio or platelet-to-lymphocyte ratio by directly incorporating metabolic status alongside immune competence. This integration of metabolic and immune information provides a more comprehensive assessment of host–tumor dynamics and may explain the strong association between GLR and OS observed in our study.

An additional noteworthy finding was the beneficial impact of prior thoracic radiotherapy. Patients with a history of radiotherapy experienced improved OS and PFS, consistent with preclinical and clinical evidence that radiation enhances immunotherapy responses by inducing immunogenic cell death, increasing antigen release, and activating dendritic cells (33). The well-described abscopal effect provides further support for combining radiotherapy with PD-1 blockade, and our results reinforce the value of multimodal therapeutic approaches in selected NSCLC patients (34).

From a clinical standpoint, GLR is highly attractive as a biomarker. It is inexpensive, reproducible, and universally accessible from routine laboratory testing, unlike PD-L1 expression or tumor mutational burden, which require specialized assays and invasive tissue sampling (35). GLR could therefore serve as a practical tool for risk stratification at baseline and potentially for longitudinal monitoring. Patients with elevated GLR may represent a subgroup at higher risk of poor outcomes who require intensified clinical surveillance, early supportive interventions, or consideration for enrollment in clinical trials evaluating novel combinations. For instance, in patients with unavailable or borderline PD-L1 expression, an elevated GLR may prompt closer follow-up intensity, earlier radiologic reassessment, or prioritization for combination or trial-based strategies rather than reliance on monotherapy alone.

The interpretation of GLR should be considered within the broader landscape of existing immunotherapy biomarkers. Established markers such as PD-L1 expression and tumor mutational burden primarily reflect tumor-intrinsic characteristics, whereas GLR captures systemic immunometabolic status at the host level. This host-centered perspective suggests that GLR may offer particular incremental value in clinical scenarios where tumor-based biomarkers are unavailable, inconclusive, or discordant with observed clinical behavior. Unlike inflammation-based indices such as the neutrophil-to-lymphocyte ratio or platelet-to-lymphocyte ratio, GLR uniquely integrates metabolic dysregulation with immune competence into a single composite metric. This distinction suggests that GLR is not intended to replace tumor-based biomarkers, but rather to provide complementary prognostic information reflecting host responsiveness to immune checkpoint blockade. In real-world settings where tissue-based biomarkers may be unavailable, inconclusive, or heterogeneous, GLR may offer a pragmatic and biologically grounded tool for baseline risk stratification. Future prospective studies incorporating multimodal biomarker frameworks—including GLR alongside PD-L1, inflammatory indices, and genomic markers—will be essential to determine its additive value and optimal clinical integration.

Finally, the translational implications of GLR extend beyond its prognostic role. By highlighting the link between systemic metabolism and immune function, GLR underscores the potential value of metabolic interventions as adjuncts to immunotherapy. Approaches aimed at optimizing glycemic control, preserving lymphocyte pools, or targeting tumor metabolic pathways may improve the efficacy of PD-1 blockade. Thus, GLR may serve not only as a clinical biomarker but also as a hypothesis-generating tool for future translational studies designed to overcome resistance to immunotherapy.

In summary, our findings identify GLR as a biologically plausible and clinically practical biomarker for patients with advanced NSCLC receiving nivolumab. Its strong association with OS, consistency with established immunotherapy trial results, and integration of metabolic and immune information highlight its potential utility in immuno-oncology.

## Limitations

This study has several limitations. First, its retrospective multicenter design introduces risks of selection bias and heterogeneity in clinical practice patterns across centers. Second, GLR was measured only at baseline, and dynamic on-treatment changes—which may offer additional prognostic or predictive insights for immunotherapy—could not be assessed due to the retrospective nature of data collection. Third, molecular correlates such as tumor mutational burden, T-cell clonality, or tumor microenvironmental characteristics were not available, limiting our ability to mechanistically validate the observed associations.

In addition, incomplete availability of established biomarkers—particularly PD-L1 expression and tumor mutational burden—precluded direct comparative or integrative analyses, limiting definitive conclusions regarding the incremental therapeutic utility of GLR relative to tumor-based or inflammation-derived biomarkers.

Another important limitation is the absence of an external validation cohort. Although the dataset included patients from 21 institutions, all centers contributed through a unified national electronic registry, which prevented the formation of an independent validation subset. Thus, while the prognostic effect of GLR remained directionally consistent across multiple clinically relevant subgroups, external validation in independent populations is required before clinical implementation. Furthermore, patients in the high-GLR group more frequently exhibited poorer ECOG performance status at baseline. Although ECOG was adjusted for in multivariate analyses, residual confounding related to baseline functional status cannot be entirely excluded.

Moreover, because GLR incorporates fasting glucose, non–tumor-related factors—including diabetes mellitus, corticosteroid exposure, metabolic syndrome, or acute inflammation—may influence its value. Baseline corticosteroid use was not uniformly documented across participating centers and therefore could not be reliably included in multivariate analyses, representing an inherent limitation of this retrospective real-world dataset. Detailed metabolic parameters (e.g., HbA1c) and comprehensive medication histories were not uniformly available across centers, limiting our ability to adjust for these confounders and representing an inherent constraint of retrospective real-world datasets. Similarly, detailed nivolumab exposure data, including exact cycle numbers, were not consistently recorded across all participating centers, precluding a reliable cycle-based exposure analysis for the entire cohort.

Finally, variability in imaging schedules and follow-up intervals across institutions may have introduced measurement noise, particularly affecting PFS assessments, despite adherence to national nivolumab monitoring guidelines. Future prospective studies with harmonized imaging protocols, standardized metabolic assessments, and longitudinal GLR measurements—as well as multicenter registries specifically designed for biomarker validation incorporating external validation cohorts, resampling techniques (e.g., bootstrapping), and calibration analyses—will be essential to confirm the reproducibility and generalizability of GLR as a prognostic biomarker.

### Clinical implications

GLR is inexpensive, reproducible, and universally available. Unlike PD-L1 expression or tumor mutational burden, it requires no specialized testing and can be obtained repeatedly from routine blood work. GLR may therefore serve as a practical stratification tool, identifying high-risk patients who warrant closer monitoring, alternative systemic strategies, or enrollment in clinical trials of novel combinations.

## Conclusion

Baseline GLR independently predicts overall survival in advanced NSCLC patients treated with nivolumab, reflecting an unfavorable immunometabolic state that compromises PD-1 blockade efficacy. Its prognostic impact on OS is consistent with the OS-driven benefit seen in pivotal immunotherapy trials. Given its accessibility and cost-effectiveness, GLR represents a promising biomarker that could complement established predictors, pending validation in prospective studies.

## Data Availability

The datasets presented in this study can be found in online repositories. The names of the repository/repositories and accession number(s) can be found in the article/supplementary material.
